# Flexibility of deployment: challenges and policy options for retaining health workers during crisis in Zimbabwe

**DOI:** 10.1186/s12960-019-0369-1

**Published:** 2019-05-31

**Authors:** Wilson Mashange, Tim Martineau, Pamela Chandiwana, Yotamu Chirwa, Vongai Mildred Pepukai, Shungu Munyati, Alvaro Alonso-Garbayo

**Affiliations:** 1 ReBUILD Consortium and Biomedical Research and Training Institute, 10 Seagrave Road, Corner Seagrave and Sam Nujoma Street, Avondale, P.O. Box. CY 1753, Causeway, Harare, Zimbabwe; 2 ReBUILD Consortium and Department of International Public Health, Liverpool School of Tropical Medicine, Liverpool, L3 5QA United Kingdom

**Keywords:** Appointment, Bonding, Deployment policies, Reappointment, Rural areas, Secondment, Socio-economic crisis, Training, Transfers, Zimbabwe

## Abstract

**Background:**

Zimbabwe experienced a socio-economic crisis from 1997 to 2008 which heavily impacted all sectors. In this context, human resource managers were confronted with the challenge of health worker shortage in rural areas and, at the same time, had to operate under a highly centralised, government-centred system which defined health worker deployment policies. This study examines the implementation of deployment policies in Zimbabwe before, during and after the crisis in order to analyse how the official policy environment evolved over time, present the actual practices used by managers to cope with the crisis and draw lessons. ‘Deployment’ here was considered to include all the human resource management functions for getting staff into posts and managing subsequent movements: recruitment, bonding, transfer and secondment. The study contributes to address the existing paucity of evidence on flexibility on implementation of policies in crisis/conflict settings.

**Methods:**

This retrospective study investigates deployment policies in government and faith-based organisation health facilities in Zimbabwe before, during and after the crisis. A document review was done to understand the policy environment. In-depth interviews with key informant including policy makers, managers and health workers in selected facilities in three mainly rural districts in the Midlands province were conducted. Data generated was analysed using a framework approach.

**Results:**

Before the crisis, health workers were allowed to look for jobs on their own, while during the crisis, they were given three choices and after the crisis the preference choice was withdrawn. The government froze recruitment in all sectors during the crisis which severely affected health workers’ deployment. In practice, the implementation of the deployment policies was relatively flexible. In some cases, health workers were transferred to retain them, the recruitment freeze was temporarily lifted to fill priority vacancies, the length of the bonding period was reduced including relaxation of withholding certificates, and managers used secondment to relocate workers to priority areas.

**Conclusion:**

Flexibility in the implementation of deployment policies during crises may increase the resilience of the system and contribute to the retention of health workers. This, in turn, may assist in ensuring coverage of health services in hard-to-reach areas.

## Introduction

Scaling up the healthcare workforce as well as improving distribution, management, performance and quality were solutions proposed to address human resources for health (HRH) crisis in the last decade [1, 2]. The inequitable distribution of HRH, especially between urban and rural areas, and the problems related to filling posts in remote areas have been identified as barriers in the provision of healthcare and the achievement of the health-related Sustainable Development Goal [3]. This is particularly prevalent in post conflict and crisis-affected settings [4, 5].

Much research has already been devoted to understanding health worker (HW) behaviour in relation to taking up and remaining in posts in rural/remote locations [6, 7]. Additionally, research has shown that the problem of mal-distribution of HRH is often made worse by the mismanagement of deployment systems [8, 9]. The term ‘deployment’ is frequently used in relation to distribution of staff, but rarely defined. More specifically, deployment is defined as ‘the movement of staff from one’s current assignment to another, to meet operational needs’ [10] or simply ‘the transfer of a person from one position to another’ [11]. In this study, however, we define deployment as a ‘bundle’ of human resource management functions for getting staff into posts and managing subsequent movements including recruitment, reappointment, bonding, secondment and transfer [12]. Within a country’s healthcare system and regulations, each of these human resource management (HRM) functions is governed by a set of policies which are sometimes official and explicit, but in other cases, they remain unwritten rules. In this paper, we explore the official policies and the informal practices that rule deployment of HWs in Zimbabwe, with the aim of reviewing the changes in deployment policies and the implementation practices during the crisis and post-crisis periods.

Zimbabwe experienced a decade of severe economic, social and political crisis between 1997 and 2008. The crisis led to a reversal of gains made in key health indicators including immunisation, maternal mortality or infant mortality [13–16] and had an important impact on the health workforce. While out-migration of HWs was reported before 1997 [17, 18], it increased substantially during the crisis leaving the staffing situation severely deteriorated with high vacancy rates which peaked between 2008 and 2009 reaching up to 43% [13, 15, 19].

Prior to 2005, the health services in Zimbabwe were delivered through facilities run separately by government, faith-based organisations (FBOs) or private sector. Government hospitals and primary healthcare facilities are managed either by the Ministry of Health and Child Care (MOHCC) or Ministry of Local and Urban Development [20]. The Zimbabwe Association of Church Related Hospitals manages church-related health providers in coordination with the MOHCC particularly for issues related to their workforce. The majority of FBO facilities operate in rural settings [21] while government run facilities cover both urban and rural areas.

In 2005, Government of Zimbabwe decided to unify health services provided by both government and FBOs. The Health Services Board (HSB) was established through the Health Act of 2004 which resulted in both government and FBO health service providers being under one central authority. Due to the enactment of the Health Services Regulations (HSR) in 2006, the deployment of government HWs employed in government and FBO facilities should be managed by MOHCC through the HSB. However, implementation of the new policy was uneven. Service contracts between MOHCC and FBOs were not implemented in all provinces simultaneously [14] which resulted in the uneven adoption of the terms comprised in the new regulation [22] particularly in those related to staff recruitment and deployment by health services managed by FBOs. This retrospective study looks at changes in deployment policies and practices in Zimbabwe’s health sector during the pre-crisis period (1980–1997), crisis period (1997–2008) and the post-crisis period (2009–2013).

There is little evidence of flexibility of these policies and practices in time of conflict or crisis [23] with the exception of post-conflict Timor Leste [24, 25]. This study sought to contribute to this evidence.

## Methods

This retrospective qualitative study was undertaken in two healthcare providers (FBOs and Government) in three largely rural districts in Zimbabwe where implementation of deployment policies and practices was explored. Data was collected through interviews with key informants involved in policy development and with managers and HWs involved in their implementation. Narratives about the life of HWs were used to generate data about the impact that changes in HRH deployment systems and practices have on their professional trajectory and experiences [26].

A total of 76 documents were extracted from published and unpublished literature and analysed using content analysis to explore the evolution of deployment policies including the rationale for changes.

The study focuses on four main HRM functions related to deployment: recruitment, bonding, transfer and secondment. We define recruitment as the process of attracting the right people and selecting and offering employment to the best candidates. We have also included here appointment, which in this study refers to the allocation of a position to a HW when entering the health workforce for the first time. We consider bonding as the formal agreement with the government comprising compulsory service that newly graduated HWs who were beneficiaries of public financial support for their studies agree to provide after graduation to compensate for the government’s investment. Transfer in this study refers to the process of moving HWs from one post to another and secondment, which is often considered as a modality of transfer, refers in this study to the temporary transfer of workers to another post for a limited period for a specific reason.

The study was undertaken in three largely rural districts of the Midlands province. The districts are referred to numerically in this paper as districts 1, 2 and 3. The major providers of healthcare in the selected districts are government and a large FBO which owns 52% of the faith-based hospitals in the country (Table 1). Government-run facilities in this study are comprised of those managed by HSB and rural district councils (RDC).

**Table 1:** Population and numbers of facilities by ownership in study districts

District	Rural population	Government-owned health facilities	FBO-owned health facilities	RDC facilities
District 1	80 351	6	4	6
District 2	175 835	10	1	15
District 3	576 362	11	9	26
Total		27	14	47

Source: compiled from MoHCW Health Facility List 2010/ ZIMSTATS 2012 Census Report

A total of 17 key informant interviews (KIIs) from national and local levels for both government and FBOs were purposively selected according to their knowledge and experience of policy development relating to deployment (Table 2).

**Table 2:** Number of key informant interviews

Organisation/sector	Sample by gender		Sample total
	Male	Female	
Public sector/HSB	8	3	11
FBO	0	3	3
Health professional associations	2	1	3
Totals	10	7	17

The overall manager and the officer responsible for HRM including deployment at facilities were selected in each study district for interviews and asked questions mainly about deployment practices. A total of 11 in-depth interviews (IDI) were carried out at both government and FBO institutions (Table 3).

**Table 3:** Distribution of IDIs with facility managers by sector and gender

Sector	Districts									Grand totals
	District 1			District 2			District 3			
	Female	Male	Total	Female	Male	Total	Female	Male	Total	
HSB	0	3	3	1	1	2	0	2	2	7
FBO	1	0	1	1	0	1	1	1	2	4
Totals	1	3	4	2	1	3	1	3	4	11

A total of 67 interviews with midwives, nurses and environmental health practitioners (42 females and 25 males) working in the three different sectors with different lengths of service (11 < 5 years; 56 ≥ 5 years) were conducted (Table 4) using the ‘life history’ technique to elicit their views and perspectives [26].

**Table 4:** Distribution of IDIs with HWs by sector and gender

Sector	Districts									Grand totals
	District 1			District 2			District 3			
	Female	Male	Total	Female	Male	Total	Female	Male	Total	
HSB	7	3	10	9	6	15	3	6	9	34
FBO	4	1	5	6	2	8	7	2	9	22
RDC	1	1	2	3	2	5	2	2	4	11
Totals	12	5	17	18	10	28	12	10	22	67

Separate topic guides for semi-structured interviews for each group were developed and used flexibly as a guide for the interviewer allowing for new emerging thematic areas introduced by interviewees. The interviews for the KIIs lasted an average of 25 min while those for managers and health workers interviews with life histories lasted between 30 and 45 min. During data collection, researchers met on daily basis to discuss the emerging themes assessing whether research questions were being answered. Saturation was achieved with the initially planned interview schedule with no need for further additional interviews [27]. All interviews were transcribed verbatim and checked for accuracy by a second transcriber. Transcriptions of the combined set on interviews were analysed using a framework approach [28]. A coding framework was initially developed based on the document review, key research questions and new themes emerging during the analysis were added. All transcriptions were coded using the developed coding framework. The data was managed using NVIVO qualitative analysis software version 10.

## Results

### Recruitment and appointment

#### Initial policies and further adaptations

Recruitment in Zimbabwe is regulated through the Public Service Regulations (PSR), 2000 sections 2 and 3/1a-d [29]. These policies envisage that recruitment should be merit-based on the applicant’s knowledge about their tasks and their ability to perform them. Appointments can be either under ‘indefinite pensionable conditions’ or ‘temporary’ to fill casual vacancies or supernumerary posts. All appointments should follow principles of equality of fair competition among candidates and need to be included in the Estimates of Expenditure approved by the Ministry of Finance each fiscal year. To confirm the suitability of new workers, they need to go through a probation period which initially was not less than 1 year after which and, if satisfactory, appointment was confirmed.

With the introduction of HSR 2006, recruitment responsibility for the health sector was shifted to the HSB [22]. Besides merit, HSR 2006 introduced criteria of professional and moral standing and considered apprenticeship for individuals less than 18 years (previously not allowed under PSR 2000) as legal to allow health professional students to undertake their required internships to complete their studies. In order to address the high attrition from the public to the private sector observed after Independence, the new regulation provided greater flexibility for dual practice allowing HSB to authorise specific workers to devote part of their time to private practice and reducing the minimum probation period from 1 year to 6 months.

#### Implementation practices

During the pre-crisis period, HWs would search for jobs on their own after training.

From 1992 to 1995 I did my nursing training as an RGN at Harare central hospital […] we were not being deployed. Each person would look for his/her own place (HW Gvt District 3).

Before the crisis, recruitment of students was done by the relevant health service providers (government or FBO), drawing from their own training schools, but it was carried out in accordance to the MOHCC/HSB standards and criteria. Despite being regulated by the same framework, church-run nursing schools had the opportunity to retain the nurses they like at their facilities after completion of training:

…the DNO [District Nursing Officer] will deploy them to us but [the nurse training school] can just say we need three here [at FBO facility] and they can even mention names preferably so and so […] (Manager FBO District 1)

However, during the crisis period (1997–2008), they had the option to choose three priority provinces, both for students who had trained in government and in FBO-run training schools. Preference listing was withdrawn during the crisis period for nurses and paramedical staff mainly to address the shortage of HWs particularly in rural areas. On the other hand, doctors were still allowed to choose, although the human resources (HR) department at MOHCC head office would intervene to ensure even deployment in areas where majority of newly qualified doctors would have chosen one hospital.

Most people (80%) would choose Harare and only 20% would choose Bulawayo but it was supposed to be 50-50. This meant that 30% of those who chose Harare were then forced to go and work in Bulawayo. To solve the issue there was a list that would come from the head office Human Resources Directorate of who would go where. (Manager Gvt District 1)

Despite the establishment of the HSB which, in the views of the government, aimed at the unification of all health services, there were some differences in the implementation of HSR 2006 between government and FBO service providers. Some managers reported having two different and parallel processes for recruiting new workers: one triggered by the HSB and another one by the specific FBO:

When we are recruiting [under the FBO process] we advertise the posts, and then people apply. When they are sent by the DNO [official HSB process] we just accept them. We sit down with them and do a bit of an interview but not as we do with those we recruit on our own because there is no option to say we want you or we don’t because the person would have been already sent to us. We then ask them to fill the assumption of duty forms and other forms which are needed. (Manager FBO District 3)

These overlapping recruitment systems with different degrees of thoroughness in terms of merit-based selection may have resulted in different recruitment outputs with a fairer system applied to these recruited through the FBO system (e.g. using interviews) as compared with these recruited through the HSB which may be more prone to patronage and weaker accountability. However, this was not substantiated by the study.

Another factor that contributed to the poor staffing situation during the crisis was the recruitment freeze introduced as a response to an over-inflated civil service [30]. However, given its key role and its already precarious staffing situation, the freeze in the health sector was temporarily relaxed to allow for limited recruitment on a periodical basis to cover urgent needs [20]. This affected equally government and FBO-run facilities.

When we have people, who qualify we wait for the unfreezing of posts. The HR [department] will allocate the posts to the provinces and provinces allocate to the districts. (National Manager Gvt)

During the crisis, the number of absconders (i.e. workers leaving the workforce without prior notice or authorisation) increased substantially. While before the crisis the process of re-appointment of absconders was long and often unsuccessful, this was adjusted after the crisis to allow for fast-tracked re-appointment to mitigate the increasing vacancy rate.

A new cadre, a primary care nurse (PCN), was introduced during the crisis to resolve staff shortages in rural areas and this cadre was meant to work at rural health centres/clinics only.

In hospital [District Hospital] it [introduction of PCNs] did not help in any way, but it helped in the rural areas, that cadre was meant to work in the rural areas. They are not supposed to work at district hospitals or other upper hospitals. (Manager Gvt District 2)

### Bonding

#### Initial policies and further adaptations

Bonding policies were first adopted in Zimbabwe through the “Health for All Action Plan” in 1986. Initially bonding arrangements were between MOHCW and professional associations [13, 17, 31] by which all new HWs were to serve generally in the public sector for a period equivalent to the period of their training. However, bonding was not mentioned in the PSR 2000 or in the HSR 2006. This policy gap may be the cause behind recent research showing inconsistent implementation of this specific policy element [20]. New regulation was introduced in 2007 by which all newly graduated nurses had their certificates withheld until they completed their bonding obligations. However, after the introduction of the recruitment freeze and the subsequent difficulties for new professionals in finding a job in the public sector, the regulation was not enforced from 2010.

#### Implementation practices

Before the crisis, bonding regulations were well enforced with bonding playing a dual role: improving retention and providing a supervised induction after the training.

Long back it [bonding] was there to retain nurses as the nurses were trained using public funds. It was also used to make sure that the nurse will be moulded through the supervision by the senior [staff] during the bonding period. (Provincial Manager Gvt).

There was change in bonding policy in 2007 when it included withholding of certificates to mitigate the increasing attrition from the public to the private sector and through out-migration. Bonding rules were the same for staff working in both government and FBO sectors especially after the creation of the HSB.

The nurses were bonded for a period equivalent to the training period and you would not get certificates for the three years you are bonded. (Provincial Manager Gvt)

There were some exceptions in the application of bonding rules within the FBO sector for ordained staff who are directly managed by the church authorities through internal church policies

Like after the training we used to know that a person will be bonded for a year before he/she can say I no longer want to be here, but for us, sisters [ordained staff], that rule doesn’t apply, we stay at a place until we are told to move. (HW Gvt District 2)

The introduction of the recruitment freeze led to discontinuation of withholding of certificates as the government could no longer guarantee employment to new graduates.

At the moment because of the shortage of vacancies and the freezing of posts, the nurses are now being given their certificates immediately after qualifying. (Provincial Manager Gvt)

Bonding for in-service training, particularly after the crisis, was perceived as fairer than for pre-service training, as it was reported by many HWs as a way of giving back to the system what the system invested in their education:

It is good in the sense that you would have been sent by the facility, so you have to render back the service, if you think of going somewhere it would not be fair that after training you will just go without rendering the service. (HW Gvt District 3)

### Transfers

#### Initial policies and further adaptations

Before 2006, transfer of civil servants was regulated through section 13 of PSR 2000. The policy reflects a compulsory top-down approach to transfer, for example stating, ‘a member may at any time without his consent be transferred by the Commission or a delegated authority from the post which he occupies to any other post in the Public Service whether the post is inside or outside Zimbabwe’ (page 16). Refusal to obey an instruction of transfer is treated as an act of misconduct. However, the regulations say that transfer should be planned to minimise discomfort to the member concerned and their family and that the member should be provided with all necessary information relating to the transfer and notified in time. Transfers cannot be used as a punitive measure except in cases of disciplinary procedures.

The PSR 2000 was replaced by HSR 2006 and in the new regulation the length of a temporary posting was left to be determined by the HSB as opposed to the initial transfer for a limited period of maximum 3 years.

#### Implementation practices

During the pre-crisis period, managers were more in control of the processes of transferring workers, but during the crisis, due to substantial problems with attrition, they were less eager to allow transfers of staff.

…as an HOD [Head of Department] I just realize that if someone moves away there will be less [fewer] nurses here so I might refuse the person to move to another facility. (Manager Gvt District 3)

These circumstances made it difficult for managers to deploy staff in a way that would improve geographical coverage. Managers also needed to balance carefully the requests from workers willing to be transferred as refusals may trigger greatly needed HRH to withdraw, abscond and go to private sector or leave the country.

Transfers were easy during the crisis because there were more vacancies than HWs seeking those posts. One [a manager] had to be reasonable; it was either you let the cadre transfer to a preferred post or the system will lose the cadre altogether. (Manager Gvt District 1)

In this regard, workers were allowed to transfer only when they found another worker in a similar position willing to exchange/swap positions.

[…] when I tried to talk to the DNO that I want to go to XXXX [Province] I was told that I should seek for a lateral transfer. I should convince somebody who is at a facility where I want to go so that he will come here, and I will go there. (HW Gvt District 1)

### Secondment

#### Initial policies and further adaptations

The PSR 2000 states that a worker may at any time, with his/her consent and at the invitation of head of Ministry or the Commission, be seconded (a temporary form of transfer) by the Commission for a period not exceeding 3 years to a post in an approved service. Terms and conditions of services for seconded workers should be governed by the contract between the worker and the service concerned. The HSR 2006 revision points out that the period of secondment will be determined by the HSB without his/her consent while the rest of the terms and conditions are as stipulated by PSR 2000.

#### Implementation practices

During the crisis, because of the challenges of transferring staff explained above, managers used secondment to fill vacant posts, but it was unpopular among HWs as the legal terms for secondment were not appropriately applied. In some cases, HWs were seconded for a longer period of time than stipulated and sometimes communication about the secondment was not provided timely and it was not clear:

I heard that the first group volunteered but as for our group we did not volunteer we were told to go. We were just told that you are going tomorrow, and we were not prepared to go. We could not even give excuses we were just told to go (HW FBO District 3)

Some key positions within the FBO health services such as medical doctors were historically staffed through internal church-related networks and were never made explicit and included in the Public Service staffing norms when the HSB was introduced. During the crisis, when even FBO facilities faced problems with mobilisation of resources from external sources, many FBOs found themselves with shortage of this specific cadre. In these cases, secondment was used to allocate physicians to these facilities.

My post is at XXX District Hospital [HSB]. When I came into the district, mission hospitals did not have posts for doctors from the government, so I was seconded here at XXX Hospital [FBO]. (Manager FBO District 1)

## Discussion

This study was designed to review the changes and flexibility in deployment policies and the implementation practices during the crisis and post-crisis periods in Zimbabwe. Much of the response to the crisis and post-crisis situations relating to deployment was in the implementation of policies and in particular to transfer and secondment. The pragmatic response of the district-level managers was to be flexible in the use of the transfer rules aiming more at retaining HWs than to ensure equitable staffing of health facilities. Here, flexibility was used in a positive way, though elsewhere flexible interpretation of rules may be seen as negative [32]. However, secondment was used as an alternative and temporary way of filling vacancies. The aim of this, in the view of managers, was to at least retain staff. Interestingly, while we found that secondment (also known as deputation in some organisations) was driven by management in our study, elsewhere it is frequently used by employees as a means of changing their place of work through “side payments” to managers [33].

Several changes in the broader context that were highly relevant to understand the evolution of HRH deployment were identified. The first was the desire to improve equitable access to health services by bringing in and coordinating the then well-resourced FBO facilities in line with government plans and funding. The introduction of a blanket recruitment freeze for the Civil Service represented a major challenge for district- and facility-level health service managers, particularly in rural areas. However, the system managed to mitigate some of the constraints by allowing flexibility on its implementation like temporarily lifting the freeze to allow recruitment and appointment for critical positions. Such exemptions for doctors and nurses were used during a lengthy recruitment freeze in Zambia in 2002 and 2004 [9]. Similarly, the policy on bonding was changed during the crisis and post-crisis periods, with for example the bonding period for nurses going from 3 to 2 years. With such a big staff shortage and a fluid labour market, the government could not afford to drive people away from the sector, so understandably they became more lenient. When even the reduced period of bonding was not being accepted by workers, the government initially used the common strategy of withholding graduation certificates during the bonding period [34] but following the recruitment freeze when jobs could no longer be guaranteed both the bonding policy and the policy of withholding of certificates were relaxed which also shows the flexibility of the system.

On the other hand, the actions—or to some extent the lack of action—of policy makers in a crisis such as that experienced in Zimbabwe can be (at least partially) explained by the fact that it was difficult or impossible for them to predict how long the crisis would last, and, based on that, it was difficult to decide what changes to make to deployment policy even when the health workforce was clearly haemorrhaging.

While the changes in response to the crisis in deployment-related policy might have been limited, adjustments and adaptations at the practice level, in particular in relation to transfer and secondment, were more prominent. In particular, the relaxation of transfer rules at the height of the crisis and the tactical use of secondment demonstrated the ability of managers’ flexibility in implementing policy—what Bossert [35] refers to as ‘decision space’—and to carry out actions to achieve their objectives, which in this case were to retain staff and to fill vacancies. Nevertheless, the bonding policies were clearly adapted at various stages of the crisis and post-crisis periods in response to the changing labour market situation. The shift on the policy of reappointment was clearly triggered by the window of opportunity as HWs who had left the sector were beginning to return in view of the apparent improvements in the overall situation. The timing of this window was fortunate, as such opportunities may be quite delayed as found by our research colleagues in Sierra Leone [36].The introduction of the PCN was also timely as it made sure posts were filled in rural areas. However, no official changes in the transfer and secondment policies were made specifically in response to the crisis and post-crisis situation.

Though the deployment objectives might have been achieved reasonably successfully in the short term, the actions of FBO managers of preventing HWs from transferring and of government managers to force staff into short-term secondments meant that in the longer term staff in both cases may have left at the first opportunity. Hence, the longer-term objective of retention would not have been achieved, illustrating the challenge of developing coherent HR strategies [37, 38]. In these situations, managers should be supported to take the initiative to solve problems but should also be supported in understanding both the short- and longer-term implications of HRM actions.

An ironic finding of this study is that although the reforms enacted through the Health Services Act of 2004 created a centralised system, in the context of crisis, district-level managers were able to push their degree of ‘decision space’ [39]—or ‘room for manoeuvre’—more than in some stable but supposedly decentralised contexts [40, 41] with regard to staff deployment. In the absence of a central response to deployment policy, this must be seen as positive behaviour. However, without support on effective HRM, some decisions made run the risk of having negative longer-term consequences. This finding is an essential lesson for Zimbabwe as it deals with the aftermath of the peak of the economic crisis, as well as potentially for other countries facing similar situations. The policy should acknowledge that decision space must be increased for deployment managers in underserved rural areas. There were some limitations to this study. Documentation on policies and interviews with high-ranking key informants was difficult to obtain. Also, different accounts of the implementation of the policies were given between managers and between health workers. However, triangulation of data types and sources was used to ensure the credibility of the findings. We had initially planned to measure the impact of changing policies using routine staffing data. However, this was not finally possible as data was not available at the national level and insufficiently complete in the study districts to provide a representative sample across the categories of HWs. Nevertheless, the multi-method approach of this study allowed for qualitative data gathered through interviews with key informants, managers and staff—in particular the life histories [26]—to provide insights on the changing staffing situation. The ‘uniqueness’ of the situation in Zimbabwe makes these results very specific and therefore not directly transferable, but they add to the knowledge about how deployment policy and practice is or is not responsive to particular crises. How the crisis affected other HR policy and practice may be different and could be the subject of further study.

## Conclusion

Flexibility in implementing deployment policies is vital especially during changing conditions created by crisis in order to improve system responsiveness, retain HWs and manage their inequitable distribution in urban and rural areas. Managers at facilities and in provinces must be given mandate and support to be able to implement the policies for the interest of the HWs and facility at large.

## Abbreviations

BRTI: Biomedical Research and Training Institute
DFID: Department for International Development (UK Aid)
DNO: District nursing officer
FBO: Faith-based organisation
Gvt: Government
HOD: Head of department
HR: Human resources
HRH: Human resources for health
HRM: Human resource management
HSB: Health service board
HSR: Health Services Regulations
HW: Health worker
IDI: In-depth interview
KII: Key informant interview
MOHCC: Ministry of Health and Child Care
MOHCW: Ministry of Health and Child Welfare
PCN: Primary care nurse
PSR: Public Service Regulation
RDC: Rural district council
RGN: Registered general nurse
